# Antioxidant Potential Evaluation at Various Stages of Black Cumin Oil Production

**DOI:** 10.3390/foods13213518

**Published:** 2024-11-04

**Authors:** Dobrochna Rabiej-Kozioł, Aleksandra Szydłowska-Czerniak

**Affiliations:** Department of Analytical Chemistry and Applied Spectroscopy, Faculty of Chemistry, Nicolaus Copernicus University in Toruń, Gagarina 7, 87-100 Toruń, Poland; d.rabiej@umk.pl

**Keywords:** *Nigella sativa* L. seeds, black cumin oils, technological processes, by-products, antioxidant capacity

## Abstract

*Nigella sativa* L. seeds and their industrial process products, oils, cake, and meal, are valuable sources of bioactive compounds with antioxidant properties. In this work, the effect of technological processes on the antioxidant capacity (AC) and total phenolic content (TPC) in the black cumin oils obtained by cold pressing and solvent extraction, as well as the by-products, were evaluated. The AC values of black cumin seeds (BCS), cold-pressed black cumin oil (BCCPO), black cumin oil extracted from seeds (BCEO-S), black cumin oil extracted from cake (BCEO-C), black cumin cake (BCC), and black cumin meal (BCM) were determined by 2,2-diphenyl-1-picrylhydrazyl (DPPH) and cupric reducing antioxidant capacity (CUPRAC) assays, whereas TPC in these samples was analyzed by the Folin–Ciocalteu (FC) method. Two applied conventional oil extraction methods, screw pressing and solvent extraction, significantly affected the AC and TPC in the obtained black cumin oils and by-products. The solvent-extracted black cumin oils revealed higher antioxidant properties (DPPH = 4041–16,500 μmol TE/100 g, CUPRAC = 1275–4827 μmol TE/100 g) than the cold-pressed black cumin oil (DPPH = 3451 μmol TE/100 g and CUPRAC = 3475 μmol TE/100 g). In addition, the oil yield (20.92–48.86%) and antioxidant properties of BCCPO (DPPH = 2933–5894 μmol TE/100 g and TPC = 135–199 mg GAE/100 g) and BCC (DPPH = 1890–2265 μmol TE/100 g and TPC = 284–341 mg GAE/100 g) closely depended on the nozzle diameters (5, 8, and 10 mm) mounted in a screw press. Although both by-products were a rich source of antioxidants, BCM had significantly lower CUPRAC (1514 μmol TE/100 g) and TPC (92 mg GAE/100 g) values than BCC (CUPRAC = 3397 μmol TE/100 g and TPC = 426 mg GAE/100 g). Nevertheless, acid hydrolysis and alkaline hydrolysis of BCM extracts significantly increased their antioxidant potential. However, the DPPH (35,629 μmol TE/100 g), CUPRAC (12,601 μmol TE/100 g), and TPC (691 mg GAE/100 g) results were higher for the BCM extract after acid hydrolysis than those for alkaline hydrolysate (DPPH = 2539 μmol TE/100 g, CUPRAC = 5959 μmol TE/100 g, and TPC = 613 mg GAE/100 g). Finally, the generated AGREEprep metrics highlighted the sustainability and the greenness of the cold pressing of oil from BCS.

## 1. Introduction

At present, black cumin (*Nigella sativa* L.), also called black seed, cumin noir, or black caraway, belongs to the Ranunculaceae family and has gained increased interest due to its health-beneficial properties, such as antioxidant, antimicrobial, antidiabetic, anticancer, anti-asthma, antihypertensive, and cytotoxic activities, mainly connected to the presence of quinone alkaloids [1,2,3,4,5,6,7]. Thymoquinone is a primary quinone with a broad spectrum of pharmacological activity in black cumin seeds. Moreover, thymoquinone possesses a predominant aroma (38.23%) and is a volatile compound (21.01%) [5,8]. Generally, *Nigella sativa* L. seeds also contain other secondary metabolites, including phenolic acids, catechins, and epicatechins, with therapeutic efficacy and potent antioxidant properties in vivo and in vitro, which can play an important role as scavengers of free radicals [9].

For this reason, black cumin seeds are widely applied in the food industry, especially in Central Europe and Western Asia, not only for oil production but also as a flavoring agent with beneficial effects on human health added to several food products, such as bread, cookies, yogurt, meat, cheese, ice cream, and pickles [2,3,5]. Notably, according to the United States Food and Drug Administration (FDA) report, black cumin is “Generally Recognized as Safe”, further enhancing its applications in the pharmaceutical and food industries [8].

On the other hand, the black cumin oil market is growing quickly and has attracted the attention of consumers looking for niche oils [10]. The popularity of such a diet requires a complete characterization of niche oils, their biomedical properties, and the variability of functional components depending on the technological and storage conditions.

It is well known that the mechanical pressing of oils from oilseeds is the oldest traditional method used to produce edible oils. Over the last few years, the cold pressing of niche oils without chemical and heat treatment fits the trend of increasing the production of natural foods with high nutritional and antioxidant properties [11]. There are different mechanical methods for oil recovery from raw materials, such as using a hydraulic press, rolling press, and screw press, which can be classified as inexpensive and safe for the environment. However, the most crucial disadvantage of the cold pressing process is lower oil yield compared with solvent extraction (over 98%). Lately, some authors [12,13,14,15,16,17,18,19,20,21] have shown that different processing conditions, such as seed roasting, press head temperature, pressure, nozzle diameters and nozzle/screw distances, rotational speed, and screw frequency, affected the yields and quality (fatty acid profiles, oxidative stability, amounts of sterols, tocopherols, phenolic compounds, volatile compounds, and total antioxidant potential) of oils obtained from different sources such as rapeseed, pistachios, hemp varieties, grape, radish, *Nigella sativa* L. seeds, walnut kernels, and coriander fruits.

In contrast, solvent extraction of oils from oilseeds using organic solvents is a quick and affordable oil extraction method commonly applied at an industrial scale that enables the recovery of the oils and lipophilic bioactive compounds present in raw materials. Regardless of the large amounts of organic solvents needed, refining processes that reduce oil quality have limited the solvent extraction method.

Significant differences in oil recoveries and the concentrations of desirable (e.g., tocopherols, phytosterols, polyphenols) and undesirable (e.g., free fatty acid, phosphatides, chlorophyll) minor components can be observed in edible oils produced by both methods—mechanical extrusion and solvent extraction [11,15,20]. Other authors [22,23] have demonstrated that the qualities of black cumin oils in terms of chemical properties like oxidative status, color, fatty acid compositions, amounts of tocopherols, phytosterols, polyphenols, and total antioxidant potential, obtained by solvent extraction, were in good agreement with mechanically pressed black cumin oils.

However, differences between minor nutritional components in niche oils, produced by both conventional extraction techniques, are still contradictory and scarce. Moreover, the need for easily available natural antioxidants, mainly present in by-products of the oil industry, is apparent. It is important to know the transfer of bioactive compounds from the oilseeds to oils and by-products, as well as changes (e.g., decomposition and dimerization) that occur in oils obtained by different extraction methods. Therefore, further studies are needed to investigate the differences in the compositions of black cumin oils produced in conventional and unconventional ways.

Besides oil extraction methods, many factors, such as the oilseed genotype and processing conditions (seed pretreatment, the water content in seeds, etc.), affected the chemical compositions of residues after the mechanical and solvent extractions of oils from black cumin seeds, called “cake” and “meal”, respectively. It is well known that black cumin meal, as residue after oil extraction, can be an outstanding source of alternative ruminant feed due to its composition: crude proteins, all essential amino acids, and lipids [24]. In addition, black cumin cake and meal also contain high amounts of antioxidant compounds [25,26,27]. The increases in antioxidant potential determined by the DPPH and ABTS methods, and the total amounts of phenolics and flavonoids in defatted black cumin meal after roasting or ultrasound pretreatment of seeds with cellulase and protease were observed by Abolghasem et al. [25].

However, many phenolic compounds present in oilseed by-products are linked by glycosidic or ester bonds to matrix components, such as cell wall polysaccharides and lipophilic structures that cannot be extracted by organic solvents. These chemical bonds can be broken, and the bound phenolic compounds can be released from oilseed residues after acid or alkaline hydrolysis. Acid hydrolysis can break glycosidic bonds, while alkaline hydrolysis can hydrolyze the ester bond and ether bond between the phenolic compounds, as well as the cell wall, to release phenolic compounds more effectively. Recently, acid and/or alkaline hydrolysis procedures have been used to extract bound phenolic compounds from different agro-wastes such as olive mill waste, *Carapa guianensis* and flaxseed cakes, shell cake of cashew nut, shell of coconut and hull of groundnut, brewery spent grains, blackberry residues, hazelnut shells, wheat straw, and orange and pitahaya peels [28,29,30,31,32,33,34,35]. Moreover, the effects of acid and alkaline hydrolysis on the total amounts of phenolics and flavonoids, as well as the antioxidant activity of methanolic extracts from *Nigella sativa* L. seeds powder, were evaluated [36].

Although a few studies have reported on the antioxidant properties of black cumin seeds and their products, to our best knowledge, the changes in the antioxidant potential throughout the black cumin chain, from seeds to oils and residues, have not been investigated. It is important to carry out a comprehensive study clarifying the effects of oil extraction methods, their conditions, and individual production stages on the antioxidant properties of raw material (*Nigella sativa* L. seeds), final products (screw-pressed and solvent-extracted oils) and by-products (cake and meal).

Therefore, the main goal of the present research was to evaluate the influence of various technological stages on antioxidant capacity (AC) and total phenolic content (TPC) in raw material—black cumin seeds (BCS), cold-pressed oil (BCCPO), solvent-extracted oils from seeds (BCEO-S) and cake (BCEO-C), and by-products—cake (BCC), and meal (BCM), measured by 2,2-diphenyl-1-picrylhydrazyl (DPPH), cupric-reducing antioxidant capacity (CUPRAC), and the Folin–Ciocalteu (FC) methods. Moreover, the impact of different nozzle diameters applied during screw pressing on oil yield and the BCCPO and BCC quality, including their antioxidant properties, was examined. Taking into account one of the interesting approaches for valorization of oil industry by-products using recovering bioactive compounds, the antioxidant properties of BCM were estimated after acid hydrolysis and alkaline hydrolysis, increasing the isolation of bound phenolic compounds.

## 2. Materials and Methods

### 2.1. Chemicals

All analytical grade chemicals used in the study were purchased from Merck Life Science (Poznań, Poland).

### 2.2. Samples

**Figure 1:**
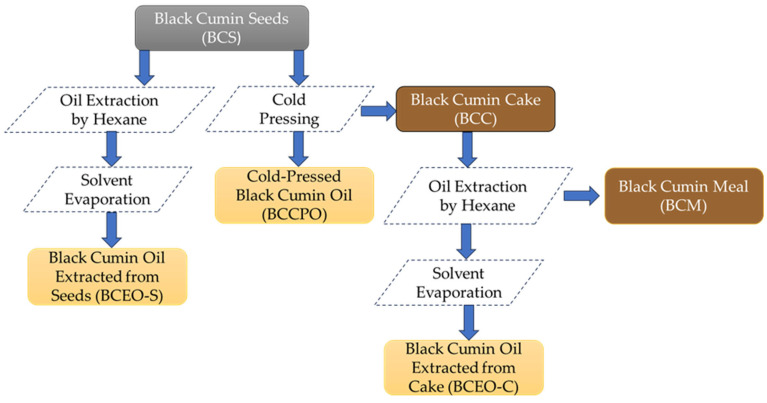
Flow chart of the studied black cumin samples.

Black cumin (*Nigella sativa* L.) seeds were kindly provided by a local vegetable oil factory. All studied black cumin samples from various steps of technological processes are presented in Figure 1.

### 2.3. Screw Pressing

Before screw pressing, the initial moisture content in black cumin seeds (6.07%) was measured with a RADWAG MA50/1.R WH moisture analyzer (Radom, Poland). Oil expression was carried out with a mechanical Komet Screw Oil Expeller CA 168 59 G—CA 59 G3 (Economic Society “MEGART” Sp. z o.o., Katowice, Poland) powered by a 1.1 kW three-phase electric motor. Screw pressing was conducted without the external heating of 250 g of *Nigella sativa* L. seeds, which were filled into a feeding hopper. The R6 (198 mm length, inner diameter of 36 mm, flight-to-flight distance of 15.5 mm) screw was used for all the experiments. In preliminary experiments, the nozzle diameter was kept constant (5 mm), while the screw speed was changed by the gearbox from positions 1 to 4 (approximate rotational speeds varied from 20 to 50 rpm). However, the same pressing speed (gearbox position = 3) was applied through all following setups, where different nozzle diameters, such as 5, 8, and 10 mm, were placed in the screw head hole. Nozzle sizes in the screw press were changed to check the influence of pressure on the BCCPO and BCC temperatures and their antioxidant properties. The temperatures of pressed oils and residues (BCC) were monitored using a FLIR C5 thermal camera (FLIR Systems, Wilsonville, OR, USA), with a resolution of 160 × 120 (19,200 pixels). Moreover, the water content in BCC samples was determined using a RADWAG MA50/1.R WH moisture analyzer (Radom, Poland).

### 2.4. Soxhlet Extraction

After grounding in a coffee grinder, BCS and BCC samples were weighted (4 × 1.00 g), fed into four prepared extraction tubes, and extracted with 10 mL of *n*-hexane in a Soxhlet apparatus fexIKA 200 CONTROL (IKA Labortechnik, Staufen, Germany). The experimental conditions (temperatures heating/cooling = 100/50 °C, number of cycles = 3, filtration time = 5 min, rev = 300 rpm) were entered into the controller program, and the extraction process was started.

A rotary evaporator was used to remove the residual solvent from oils. The oils extracted from black cumin seeds (BCEO-S) and black cumin cake (BCEO-C) were stored in the refrigerator until further analysis.

### 2.5. Determination of Oil Yield and Oil Content in Black Cumin Cake

The oil mechanical extraction yield was measured according to the procedure used by Deli et al. [21] with some modifications. The pressed black cumin oils were placed for 24 h in the refrigerator to allow foreign materials to settle. Next, the oils were filtered to remove other fine particles. The oil yield was calculated using Formula (1), as follows:

(1) $$oil\;yield\;[\%]=\frac{m_{BCCPO}}{m_{BCS}}\times 100$$

where *m_BCCPO_*—weight of pressed oil and *m_BCS_*—weight of used seeds.

The oil content in black cumin cake (BCC) was determined by the Soxhlet extraction method conducted in apparatus fexIKA 200 CONTROL (IKA Labortechnik, Staufen, Germany). The mixture of oil and *n*-hexane was separated using a rotary evaporator under vacuum. The oil obtained after evaporation was weighed and the oil in BCC was calculated using Formula (2), as follows:

(2) $$oil\;in\;cake\;[\%]=\frac{m_{BCEO-C}}{m_{BCC}}\times 100$$

where: *m_BCEO-C_*—weight of extracted oil from cake; *m_BCC_*—weight of used cake.

### 2.6. Determination of Antioxidant Properties

#### 2.6.1. Preparation of Methanolic Extracts

A total of 3.00 g of solid ground samples (BCS, BCC, and BCM) were extracted with 10 mL of methanol in the Erlenmeyer flasks, while oil samples (2.00 g) were extracted with 5 mL of methanol in 10 mL test tubes. The traditional extraction of antioxidants from all investigated samples was carried out for 30 min at an ambient temperature in the dark using an orbital shaker (SHKA25081 CE, Labo Plus, Warszawa, Poland). Then, the oil mixtures were frozen for 30 min, whereas BCS, BCC, and BCM mixtures were centrifuged (MPW-54, MPW MED. INSTRUMENTS, Warszawa, Poland) to separate methanolic extracts from samples. Extractions were repeated three times, and the combined extracts were transferred quantitatively into glass bottles.

Additionally, 80% methanolic BCM extract was prepared for hydrolysis according to the procedure used by Siger et al. [37]. Briefly, 5.00 g of defatted BCM was extracted with 50 mL of 80% methanol in a 100 mL Erlenmeyer flask for 30 min in the dark. Then, the obtained mixture was centrifuged (MPW-54, MPW MED. INSTRUMENTS, Warszawa, Poland), and the extraction procedure was repeated in triplicate. The extract was concentrated under reduced pressure (Laborota 4003, Heidolph Instruments, Schwabach, Germany) to a volume of 50 mL.

#### 2.6.2. Hydrolysis of Black Cumin Meal Extracts

**Figure 2:**
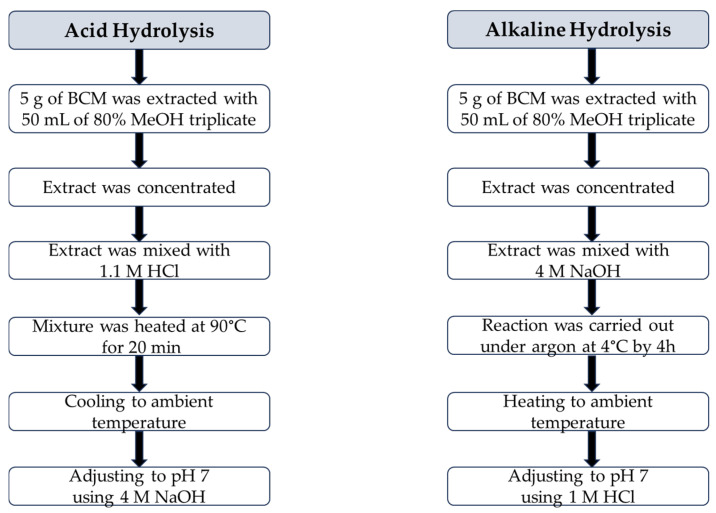
Flow chart of the procedures of acid hydrolysis and alkaline hydrolysis of black cumin meal extract.

Acid hydrolysis and alkaline hydrolysis of 80% methanolic BCM extract were conducted according to a slight modification of the procedures proposed by Siger et al. [37], as presented in Figure 2.

##### Acid Hydrolysis

A total of 19 mL of 80% methanolic BCM extract was mixed with 23 mL of 1.1 M HCl in a round-bottom flask equipped with boiling stones, a thermometer, and a reflux condenser. The reaction mixture was heated at 90 °C for 20 min. Next, the sample was cooled to an ambient temperature and neutralized to pH 7 using 4 M NaOH.

##### Alkaline Hydrolysis

A total of 19 mL of concentrated BCM extract was introduced into a round-bottom flask and mixed with 23 mL of 4 M NaOH. The reaction was carried out under an inner gas (argon) atmosphere at 4 °C by 4 h. Then, the mixture was heated to an ambient temperature and adjusted to pH 7 using 1 M HCl.

#### 2.6.3. Antioxidant Capacity and Total Phenolic Content

The AC of all prepared extracts and hydrolysates were determined by two modified spectrophotometric methods such as DPPH and CUPRAC, while the TPC in these samples was analyzed using the Folin–Ciocalteu (FC) assay. Our previous work [38] described these chosen analytical procedures in detail.

The DPPH and CUPRAC results were expressed as μmol of Trolox equivalents per 100 g of sample (μmol TE/100 g), and the TPC was expressed as mg of gallic acid equivalents per 100 g of sample (mg GAE/100 g).

##### DPPH Method

Briefly, 0.02–0.10 mL of prepared sample extracts was pipetted into glass cuvettes and brought to a volume of 2.00 mL with methanol. Then, 0.50 mL of DPPH methanolic solution (304.0 μmol/L) was introduced to each cuvette and the obtained mixtures were mixed thoroughly. After 15 min, the absorbance was measured at 517 nm against a reagent blank (2.00 mL of methanol and 0.50 mL of DPPH methanolic solution).

The calibration curve, y = (654.97 ± 8.02)x − (1.57 ± 0.03), generated for working solutions of Trolox in methanol between 0.02 and 0.10 µmol/mL with a determination coefficient (R^2^) = 0.9952 was applied to the DPPH results calculation.

##### CUPRAC Method

In brief, 0.02–0.075 mL of prepared extracts and 2 mL of each solution as follows: CuCl_2_ (0.01 mol/L), neocuproine (0.0075 mol/L), and ammonium acetate buffer (pH = 7), were transferred into 10 mL volumetric flasks and made up to volume with redistilled water. The obtained solutions were left in darkness at room temperature for 30 min and the absorbance was measured at 450 nm against a reagent blank containing all reagents except extract.

The CUPRAC of each extract was calculated using the generated calibration curve, y = (13.02 ± 0.67)x + (0.09 ± 0.01), with R^2^ = 0.9973 in the Trolox concentration range between 0.01 and 0.07 μmol/mL.

##### Folin–Ciocalteu Method

Briefly, 0.05–0.50 mL of prepared extract was transferred into 10 mL calibration flask, and 0.5 mL of FC reagent added and shaken for 3 min. Next, 1 mL of saturated Na_2_CO_3_ solution was added and made up to the mark with redistilled water and kept in darkness for 60 min. After the centrifugation of solutions (speed = 5800 rpm for 15 min, using centrifuge MPW-54, MPW MED. INSTRUMENTS, Warszawa, Poland) the absorbance at 765 nm was measured against a reagent blank. 

The calibration plot, y = (168.61 ± 4.02)x + (0.003 ± 0.0002), was linear (R^2^ = 0.9984) in the concentration range between 7.10 × 10^−4^ and 5.68 × 10^−3^ mg/mL for gallic acid methanolic solutions.

### 2.7. Determination of Peroxide Value

The peroxide value (PV) of the BCCPO samples was determined iodometrically according to the official procedure ISO 3960:2017 [39]. The PV was expressed as milliequivalents of active oxygen per kilogram of oil (meq O_2_/kg).

### 2.8. Statistical Analysis

The oil yield and quality parameters for each sample were conducted five times within one day, and the obtained results are presented as the mean value of five replicates ± standard deviation (SD). A one-way analysis of variance (ANOVA), followed by the post hoc Duncan test, was performed to analyze the significant differences between data (*p* < 0.05). Furthermore, the Pearson correlation test was applied to determine the correlations between TPC and AC of black cumin samples from the different technological stages analyzed by various analytical methods. Statistical analysis of the data were performed using the IBM SPSS ver. 9 PS IMAGO PRO Academic (institutional license purchased by the Nicolaus Copernicus University in Toruń, Toruń, Poland). Additionally, to assess and contrast the greenness of both procedures used for oil extraction, such as cold pressing and solvent extraction, the AGREEprep software (v. 0.91) (Analytical GREEness for Sample Preparation, available for free [40]) was employed.

## 3. Results and Discussion

### 3.1. Effect of Oil Extraction Methods on Antioxidant Capacity of Black Cumin Oils and Its By-Products

The AC and TPC results of *Nigella sativa* L. seeds (BCS), oils extracted from them (BCCPO, BCEO-S, BCEO-C), and by-products (BCC and BCM) determined by the modified DPPH, CUPRAC, and FC assays are listed in Table 1.

The Duncan test indicated there were significant differences in the AC and TPC values of samples at different stages of the technological oil production process. These discrepancies suggest that the oil extraction method and oil source (raw material—BCS—) significantly affected the amounts of bioactive compounds with antioxidant potential in the obtained oils and residues (cake—BCC—and meal—BCM—). Moreover, one possible explanation of the AC variability, determined by two analytical methods, is the fact that antioxidants present in the studied samples acted through the following different mechanisms: (1) scavenging of DPPH radicals (DPPH assay) and (2) formation Cu(I)-neocuproine (Nc) chelate as a result of the redox reaction of chain-breaking antioxidants with the CUPRAC reagent (Cu(II)-Nc) (CUPRAC assay). The chosen antioxidant assays also differ in affinities toward hydrophobic and hydrophilic antioxidants. The DPPH method uses radicals dissolved in organic media and thus has a higher affinity toward lipophilic than hydrophilic antioxidants, while the CUPRAC method is suitable to assess the AC of both lipophilic and hydrophilic antioxidants.

It can be noted that all investigated samples contained antioxidants with a higher ability to scavenge the chromogenic DPPH radicals (DPPH = 1219–16,500 µmol TE/100 g) than generate the colored chelate of Cu(I)-Nc (CUPRAC = 871–4827 µmol TE/100 g). Unexpectedly, untreated raw material (BCS) had the lowest DPPH (1219 µmol TE/100 g) and CUPRAC (871 µmol TE/100 g) values among all samples. In contrast, the TPC (182 mg GAE/100 g) in BCS was about 2–3 times higher than the amounts of phenolic compounds in the final residue of the technological process (TPC = 92 mg GAE/100 g in BCM) and oil extracted from cake (TPC = 70 mg GAE/100 g in BCEO-C).

Other authors [41,42] reported a similar TPC in different extracts of black cumin seeds (66.4–173.7 mg GAE/100 g in 50–100% methanolic extracts, 292.5 mg GAE/100 g in 70% methanolic extract, and 264.8 mg GAE/100 g in water extract), whereas the concentration required to scavenge 50% of DPPH radicals ranged between 372 and 432 mg ascorbic acid equivalent/g, 46.3 µg/mL, and 51.9 µg/mL for 70% methanolic and water extracts, respectively. Furthermore, Egyptian organic black cumin seeds had lower DPPH (45 mg TE/g) and ABTS (80 mg TE/g) values than defatted black cumin meal obtained after roasting (DPPH = 43–90 mg TE/g, ABTS = 80–120 mg TE/g) and the ultrasound-assisted enzymatic (DPPH = 70–120 mg TE/g, ABTS = 79–149 mg TE/g) treatment of seeds [25]. Specifically, heating and ultrasonication with the enzymatic pre-treatment of black cumin seeds increased the TPC in meal samples (TPC = 31 mg GAE/g, 32–40 mg GAE/g, 50–110 mg GAE/g, for untreated seeds and meals obtained from seeds treated with roasting, enzymes, and ultrasonication, respectively). It is worth highlighting that Egyptian organic black cumin seeds and meals contained a significantly higher TPC than our BCS and BCM samples. Similarly, methanolic (70 and 100%) and ethanolic (70 and 100%) extracts of black cumin seeds before (TPC = 13.50–19.43 mg GAE/g) and after microwave radiation (TPC = 14.92–25.76 mg GAE/g), as prepared by Hossen and Ali [43], were richer sources of phenolic compounds than the *Nigella sativa* L. seeds analyzed in this work. The microwave pretreatment of black cumin seeds for 1–3 min also caused an apparent increase in DPPH radical scavenging activity (35.25–70.51% and 38.65–82.72% for untreated and treated seeds, respectively).

The differences in the TPC among the various black cumin seeds can be due to genetic and environmental factors, the agricultural techniques applied, the technological processes (seed conditioning) and the analytical sample preparation (solvent polarity for phenolic extraction). These results suggest that bioactive compounds, such as phenolic acids, flavonoids, coumarins, and lignins naturally present in BCS, which are linked to cellular components, are not capable of scavenging the DPPH radicals, generating colored Cu(I)-Nc complex and reacting with the FC reagent. However, oil extraction parameters (crushing, rise in temperature, solvent, etc.) cause the breakage of cellular components and release into the cell medium of the phenolic compounds that were naturally retained. Moreover, new compounds with high antioxidant activities may be formed during thermal processing [44].

Therefore, oil recovery methods from BCS greatly affected the AC and TPC in the obtained black cumin oils. Significant differences in DPPH, CUPRAC, and TPC values were observed between oil samples mechanically pressed and extracted with *n*-hexane from BCS and BCC (Table 1, Duncan test). As can be seen, the highest amounts of antioxidants that can react with the DPPH radicals, Cu(II)-Nc, and FC reagents were found in BCEO-S (DPPH = 16,500 µmol TE/100 g, CUPRAC = 4827 µmol TE/100 g, and TPC = 292 mg GAE/100 g). Consequently, the BCEO-C yielded approximately four times lower AC values (DPPH = 4041 µmol TE/100 g and CUPRAC = 1275 µmol TE/100 g) and TPC (70 mg GAE/100 g) than the BCEO-S (Table 1). However, BCCPO was a richer source of TPC (259 mg GAE/100 g) and antioxidants capable of reducing the Cu(II)-Nc complex (CUPRAC = 3475 µmol TE/100 g) than BCEO-C. Nevertheless, cold-pressed oil (BCCPO) had significantly lower DPPH radical scavenging activity (3451 µmol TE/100 g) compared to the DPPH results (16,500 and 4041 µmol TE/100 g) for oils (BCEO-S and BCEO-C) extracted by *n*-hexane from BCS and BCC, respectively.

These results confirm that the amount of antioxidant compounds in black cumin oils depends on the chosen extraction method, extraction conditions, and plant material. The extraction of oils rich in antioxidants requires disrupting the membrane matrix of oilseeds and their release into the oil. In the case of the solvent extraction technique, an essential aspect is the interaction between the solvent and antioxidants, and the solvent’s ability to penetrate the plant material efficiently. It can be noted that during solvent extraction, more antioxidants, including liposoluble ones, were dissolved in *n*-hexane and released into extracted black cumin oils, enhancing their antioxidant properties. This suggests that the solvent’s ability for antioxidant extraction was more effective than an increase in temperature and pressure during the mechanical pressing method, which can change the oil’s viscosity and facilitate the solubility of antioxidants in BCCPO. Nevertheless, cold pressing is a green extraction method because it takes place without organic solvents but does not effectively release bioactive compounds with an antioxidant potential from BCS into oil.

On the contrary, Albakry et al. [22] determined a higher DPPH (IC_50_ = 2.98 mg/mL), ABTS (IC_50_ = 4.06 mg/mL), and TPC (347.12 mg GAE/kg) in black cumin oil after cold pressing than DPPH (IC_50_ = 4.01 mg/mL), ABTS (IC_50_ = 6.23 mg/mL), and TPC (314.08 mg GAE/kg) in solvent-extracted black cumin oil. However, black cumin seed oil obtained with supercritical fluid extraction was the richest source of antioxidant compounds (IC_50_ = 1.43 and 2.69 mg/mL for DPPH and ABTS assays, respectively, and TPC = 400.02 mg GAE/kg). Similarly, the antioxidant power, analyzed by a DPPH assay, and the concentration of total phenols were the highest in cold-pressed black cumin oil (DPPH = 78.45% and TPC = 36.05 mg GAE/kg), followed by Soxhlet-extracted oil (DPPH = 65.58% and TPC = 21.44 mg GAE/kg), and the lowest in black cumin oil after microwave-assisted extraction (DPPH = 61.69% and TPC = 15.19 mg GAE/kg) [11]. Moreover, the crude oil hot-pressed from black cumin seeds had a higher TPC (600 mg GAE/kg) and DPPH inhibition (90%) compared with the TPC (300 mg GAE/kg) and DPPH (59%) of the solvent-extracted oil from black cumin cake [6]. In addition, concentrations of phenolic compounds and the DPPH scavenging capacity of cold-pressed black cumin oils from six different batches ranged between 1.02 and 1.40 mg GAE/g and 76.43–83.52 µmol/100 µmol [45]. According to our previous report [4], the DPPH (226.8–790.1 µmol TE/100 g), ABTS (385.9–1465.0 µmol TE/100 g), CUPRAC (975.3–19,823.3 µmol TE/100 g), and FC (168.1–643.7 µmol TE/100 g) values for seven commercial cold-pressed black cumin oils purchased in shops on the Polish market differed significantly. 

Interestingly, black cumin oils recovered from seeds by other authors [5,6,11,22,46] using different extraction methods exhibited significantly lower amounts of total phenolic compounds (TPC = 15.19–955.77 mg GAE/kg) compared with TPC (259 and 292 mg GAE/100 g) in our BCCPO and BCEO-S samples, respectively. 

It is noteworthy that two major by-products, BCC and BCM, coming directly from the screw press and additionally undergoing *n*-hexane extraction, respectively, were rich sources of antioxidants. Insignificant differences in DPPH results were observed between BCC, BCM, and BCCPO (Table 1, Duncan test). Moreover, CUPRAC values for BCC and BCCPO did not differ significantly. However, BCM contained significantly lower amounts of antioxidants reacting with Cu(II)-Nc (CUPRAC = 1514 µmol TE/100 g) and FC reagents (TPC = 92 mg GAE/100 g) than BCC (CUPRAC = 3397 µmol TE/100 g and TPC = 426 mg GAE/100 g). Probably during solvent extraction, more antioxidants, including phenolic compounds, were released from BCS into the solvent and enhanced the antioxidant properties of BCEO-S. On the contrary, the highest amount of TPC (426 mg GAE/100 g) was determined in the obtained BCC by-product. One hypothesis for this phenomenon is that crushing and the heat generated during the mechanical pressing of BCS altered the structure of the associated bioactive components, resulting in increased levels of phenolic compounds, which did not escape into BCCPO but remained in BCC.

For comparison, a significantly lower TPC (1.44 mg GAE/100 g) in black cumin press cake was found by Tarasevičienė et al. [26], while black cumin meal contained free (116.83 mg/kg), esterified (40.58 mg/kg), and insoluble-bound (209.81 mg/kg) total phenolic acids [27].

Additionally, a Pearson correlation test was performed to correlate the antioxidant properties with the number of phenolic compounds in black cumin samples from different technological stages, from seeds to oils and residues (Table 1). There were no significant (*p* > 0.05) correlations between DPPH, CUPRAC, and TPC in any of the studied black cumin samples (correlation coefficients, r = 0.2439, *p* = 0.6414, r = 0.7273, *p* = 0.1014, and r = 0.7461, *p* = 0.0885 for DPPH-TPC, CUPRAC-TPC, and DPPH-CUPRAC, respectively). The lack of correlation between DPPH and TPC suggests that not only phenolics, but also other bioactive compounds can contribute significantly or synergistically to the broad DPPH radical-scavenging activity of black cumin samples from different technological processes. In contrast, the presence of phenolics in these studied samples can reduce Cu^2+^ to Cu^+^ by donating an electron and contributes more effectively to their antioxidant potential determined by the CUPRAC method. Therefore, different bioactive compounds may have contributed differently to the overall AC. Generally, technological processes, including thermal processing, are recognized as one of the major factors that can beneficially or negatively affect AC by the destruction or changes in phenolic compounds with higher antioxidant properties.

Positive correlations between DPPH and TPC (R^2^ = 0.817), ABTS and TPC (R^2^ = 0.567), and DPPH and ABTS (R^2^ = 0.798) of black seed oil hexane extracted from roasted and ultrasound-assisted, enzymatically treated black cumin seeds and defatted black seed meal were also found by Mashhadi Abolghasem et al. [25].

The AC and TPC results indicated that *Nigella sativa* L. seeds were a valuable source of niche black cumin oils and residues after mechanical pressing and solvent extraction, possessing strong antioxidant properties.

### 3.2. Effect of Screw Pressing Conditions on Quality and Antioxidant Properties of Cold-Pressed Black Cumin Oil and Black Cumin Cake

It is well known that cold pressing is an environmentally friendly process carried out quickly without harmful chemicals, allowing the production of high-quality crude oils which are generally highly stable. The ecological aspect and application of mechanical extrusion as a continuous process in the oil industry were the reasons for evaluating the parameters of screw pressing on the quality and antioxidant properties of the final product and residue called BCCPO and BCC.

#### 3.2.1. Temperature Monitoring

**Figure 3:**
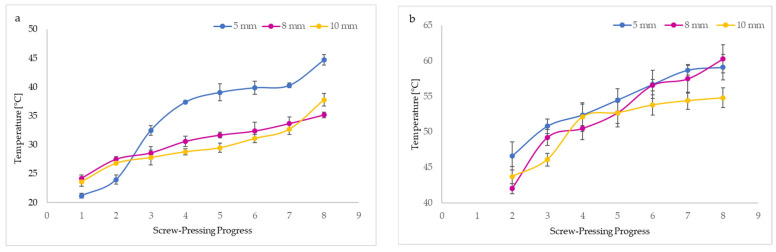

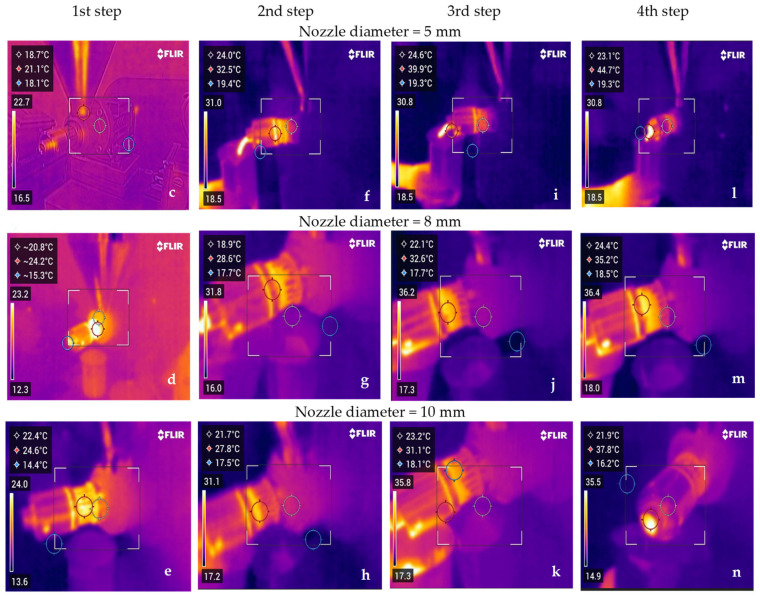
Effect of nozzle sizes in screw press on temperatures of black cumin oil (**a**) and black cumin cake (**b**) and images of temperature changes during pressing recorded by a thermal camera at the beginning of pressing (1st step) (**c**–**e**), during pressing (2nd (**f**–-**h**) and 3rd (**i**–**k**) steps), and at the end of pressing (4th step) (**l**–**n**).

Figure 3 demonstrates the progressive increase in the temperature of the oil (BCCPO) and residue (BCC) during the mechanical pressing of untreated seeds (BCS). It can be noted that nozzle diameters used in the screw press affected the temperatures of BCCPO and BCC. The oil temperatures during pressing ranged between 21.2 and 44.7 °C, whereas the BCC temperatures reached 42.0 to 60.3 °C. The temperature enhancement was probably caused by friction generated inside the screw press. Unquestionably, BCC from different pressing steps had about 2-fold higher temperatures than oil (Figure 3). Interestingly, the highest temperatures of BCCPO and BCC were recorded when the 5 mm nozzle diameter was used. However, changing the nozzle size to 10 mm resulted in the lowest oil and press cake temperatures. The smaller nozzle size caused a rise in temperatures of oil and cake as a result of the increase in friction produced in the applied screw press and the longer residence time of pressed seeds in the continuous system. The results obtained confirm that the temperatures of BCCPO and BCC depend directly on thermal transfer. In the first stage of mechanical pressing, using a screw press with a 5 mm nozzle size, the oil showed the lowest temperature (21.2 °C) and the cake the highest (46.6 °C). However, the same screw press parameters caused a sudden increase in oil temperature during the second stage of pressing (Figure 3a). As expected, the BCC obtained by mechanically pressing using smaller nozzle diameters of 5 and 8 mm had high but similar temperatures in the last stages of this process (Figure 3b).

Our findings were consistent with a previous study that reported the highest temperatures (58.33–63.67 °C) of walnut oils pressed from walnut kernels at different rotational speeds (30, 50, 70, and 90 rpm) occurred when the minor nozzle diameter (6 mm) was mounted in a screw press. Lower and similar oil temperatures (38.33–55.33 °C) were observed for the larger nozzle sizes (8 and 10 mm), where the same rotational speeds of the screw press applied [19]. Moreover, the change in nozzle diameter from 6 mm to 8 nm and 10 mm reduced the temperatures of cold-pressed rapeseed oils from 58 to 38 °C [12].

#### 3.2.2. Oil Yield and Cake Residual Oil

The yields of oils obtained from untreated BCS using different nozzle diameters in a screw press and the quality parameters of BCC as a by-product of this process are presented in Table 2.

The Duncan test indicated that black cumin oil yield significantly decreased when increasing the nozzle size mounted in a screw press used to produce BCCPO samples. The oil yield (48.86%) for a 5 mm nozzle size was above two times higher than this (20.92%) after pressing with a 10 mm nozzle diameter. Conversely, the highest cake mass (790.76 g/kg seeds) with the highest oil percentage (28.79%) in a BCC residue was found after oil pressing with a 10 mm nozzle diameter. However, the lowest cake weight (511.40 g/kg seeds) contained the lowest oil content in BCC (23.59%) obtained from a screw press having the lowest nozzle size (5 mm). This suggests that the higher pressure exerted by the smaller 5 mm nozzle size increased temperature and allowed for a higher oil recovery from BCS, thus resulting in a lower oil percentage in the corresponding residual BCC and a lower mass (Figure 3 and Table 2). Our results support that nozzle diameter significantly affected the BCCPO yield, oil content, and weight of the BCC. In contrast, water volumes (6.36–6.70%) in the three BCC samples obtained after changing nozzle diameters from 5 to 10 mm in a screw press did not differ significantly. Most importantly, an increase in pressure and temperature disrupted the cell structure, causing protein denaturation and changes in seed lipoprotein membranes, which reduced the viscosity and surface tension of oil and enhanced the thermal movement of oil molecules, resulting in improved oil dissolution and facilitating oil release. Moreover, endogenous wall-degrading enzymes were probably active during the mechanical pressing process, which, together with higher temperatures, resulted in a higher oil yield.

The obtained results of oil yield corroborate those of Deli et al. [21], which found a decrease in oil yield from *Nigella sativa* L. seeds (13.01–22.27%, 8.73–18.58%, and 13.46–19.05%) with an increase in the nozzle size (6, 10, 12 mm) and rotational speed (21, 54, 65, 98 rpm). However, using similar nozzle diameters, these authors observed significantly lower black cumin oil yields (8.73–22.27%) compared to the yields (20.92–48.86%) for BCCPO samples. Apart from mechanical pressing parameters, one possible explanation for these discrepancies can be the oil content in black cumin seeds depending on their origin, variety, and atmospheric conditions during vegetation (temperature, salinity, water stress, etc.).

Moreover, the use of the 6 mm nozzle size in a Bracco screw press resulted in a higher yield of hemp oil (262.6 g/kg seeds) and, consequently, lower cake weight (737.4 g/kg seeds) in comparison with the larger 8 mm nozzle size causing a decrease in oil yield (232.0 g/kg seeds) and an increase in cake weight = 768.0 g/kg seeds) [16]. The hemp oil yield reduction (270–285 mL, 250–275 mL, and 235–260 mL) and increase in cake residual oil (9.96–12.10%, 10.22–13.71%, and 10.42–14.16%) with an increase in nozzle sizes (6, 9, and 12 mm) were also reported by Aladić et al. [15]. Similarly, the negative impact of nozzle diameters on oil recoveries from rapeseeds (6.28–24.28%, 22.77–29.52%, and 22.35–27.45% for 12, 10, and 8 mm nozzles, respectively), radish seeds (10.0–14.5%, 12.0–15.5%, and 13.5–17.5% for 12, 10, and 8 mm nozzles, respectively), grape seeds (708–917 g, 787–956 g, and 789–987 g for 12, 10, and 7 mm nozzles, respectively), and walnut kernels (0.12–0.28, 0.15–0.31, and 0.33–0.41 kg oil/kg kernel for 10, 8, and 6 mm nozzles, respectively), was observed by other authors [13,17,18,19]. On the contrary, higher pistachio and coriander oil yields (63.3–66.4% and 30.88–65.87%, respectively) were obtained when wider nozzle diameters (6 and 8, 9, 10 mm, respectively) were employed compared to 3 mm (46.5–50.4%) and 5, 6, 7 mm (55.04–65.02%) [14,20].

#### 3.2.3. Antioxidant Properties of Black Cumin Oil and Black Cumin Cake

The nozzle diameter used in a screw press had a significant effect on the antioxidant properties of the BCCPO and BCC. As shown in Table 2, the amounts of antioxidants capable of scavenging DPPH radicals and TPC in BCCPO samples increased with the increase in nozzle size. The DPPH (5894 µmol TE/100 g) and TPC (199 mg GAE/100 g) values were the highest for oil pressed with the widest nozzle diameter (10 mm), while the lowest DPPH (2933 µmol TE/100 g) and TPC (135 mg GAE/100 g) were found in oil obtained after replacing with a 5 mm nozzle. This suggests that antioxidant compounds in screw-pressed black cumin oils are sensitive to high temperatures. Possible thermal degradation of specific antioxidants with the ability to scavenge DPPH radicals and reduce the FC reagent took place in oil extruded using smaller nozzle diameters (5 and 8 mm) due to the generation of higher temperatures during the screw pressing process (Figure 3).

Interestingly, increasing nozzle size by 3 mm resulted in about 60% higher DPPH value (from 2933 to 4880 µmol TE/100 g for 5 and 8 mm, respectively), while a 2 mm increase in the nozzle diameter to 10 mm enhanced the DPPH of BCCPO only by 20% (DPPH = 5894 µmol TE/100 g). However, insignificant differences in TPC results (135 and 145 mg GAE/100 g) were observed for oils pressed with nozzle sizes of 5 and 8 mm, while BCCPO contained approximately 1.5 times higher amounts of phenolic compounds (199 mg GAE/100 g) when pressing was carried out at a nozzle size of 10 mm.

In addition, the positive and linear correlation (r = 0.8484, *p* = 0.3551) between DPPH and TPC in black cumin oils screw-pressed using different nozzle diameters suggests that the phenolic compounds transfer to oils was responsible for the DPPH radical scavenging activity.

Unexpectedly, the lowest content of primary oxidation products—hydroperoxides (PV = 74.92 meq O_2_/kg) was determined in oil extruded with the smallest nozzle size (5 mm), generating the highest oil temperature (Figure 3 and Table 2). Nevertheless, there was a significant increase in oil hydroperoxides after changing nozzles with higher diameters (79.77 and 84.38 meq O_2_/kg for 8 and 10 mm). This progressive reduction in PV with a decrease in nozzle diameters and increased oil temperatures can be explained by the thermal degradation of highly unstable primary intermediate oxidation products and their transformation to secondary oxidation products such as aldehydes, ketones, hydrocarbons, alcohols, and esters.

In other cold-pressed oils obtained from hemp (*Cannabis sativa* L.) seeds, radish seeds, and rapeseeds, processing parameters, such as nozzle size (6–12, 8–12, and 6–10 mm, respectively), temperature (60–100 and 40–80 °C), and screw frequency (20–40 and 40–60 Hz) did not significantly affect their peroxide values [12,15,18]. The average PV results of all experimental runs were 1.95 mmol O_2_/kg, 0.46 mmol O_2_/kg, and 1.88 meq O_2_/kg for cold-pressed hemp, radish, and rapeseed oils, respectively.

It is noteworthy that the PV results (74.92–84.38 meq O_2_/kg) determined in the present study for fresh black cumin oils pressed from BCS using different nozzle diameters (5, 8, and 10 mm) were significantly higher than these (PV = 5.09 and 20.15 meq O_2_/kg) obtained by other authors for cold-pressed black cumin oils [22,23]. However, Tarasevičienė et al. [26] reported about two times higher levels of peroxides (PV = 143.1 meq O_2_/kg) in black cumin oil cold-pressed from *Nigella sativa* L. seeds originating from Egypt. Moreover, the amount of the primary oxidation products in cold-pressed black cumin oils commercially available in the Polish market varied from 21.36 to 123.77 meq O_2_/kg [4]. Also, black cumin oils extruded by hydraulic pressing and screw pressing from untreated *Nigella sativa* L. seeds purchased from Egypt and India had a high PV ranging between 65.4 and 82.5 meq O_2_/kg [47,48]. Somewhat lower PV results (11.6 to 71.6 meq O_2_/kg) were found by Dąbrowski et al. [2] for *n*-hexane-extracted black cumin oils from seeds originating from four countries (India, Egypt, Syria, and Poland). Interestingly, amounts of hydroperoxides (PV = 8.00–84.00 meq O_2_/kg) in black cumin oils extracted from convection roasted seeds and superheated steam-roasted seeds at temperatures varied from 150 to 250 °C for 10, 15, and 20 min and were lower than those obtained in oil from unroasted seeds (57.33 meq O_2_/kg) [49]. These PVs also significantly declined as the roasting temperature and time increased because the heat treatment of black cumin seeds decreased the thymoquinone content in the obtained oils. Some authors suggest that the high PV results for black cumin oils can be related to the large amounts of thymoquinone in these oils [2,47]. This major component of crude black cumin oils belongs to quinone compounds and, as an electron acceptor, possesses oxidizing properties. Thymoquinone can probably act as an interfering substance during PV analysis, leading to an overestimation of the PV results. This confirms that BCCPO pressed with a 5 mm nozzle had the highest temperature and lowest PV, probably due to a lower thymoquinone concentration. On the other hand, higher temperatures during screw pressing can deactivate the enzymes in BCS, and oils revealed lower PVs.

Some possible explanations for these discrepancies between the PV results are the variance in cultivars of black cumin seeds, the country of their origin, the cultivation conditions, and the methods and parameters of oil extraction.

Additionally, from the results listed in Table 2, all BCC samples revealed a DPPH scavenging activity, which increased with the increase in nozzle diameters. The amounts of antioxidant compounds in the obtained residues after screw pressing able to scavenge DPPH radicals were 1.5–2.5 lower than those in black cumin oils. This suggests that significant contents of hydrophobic antioxidants with DPPH scavenging activity were transferred from the BCS to oils after mechanical pressing, although the high content of these bioactive compounds remained in the by-product, namely BCC (DPPH = 1890–2265 µmol TE/100 g). Similarly to the scavenging activity of oils, these antioxidants present in the BCC samples were vulnerable to temperature because the highest DPPH found for BCC had the lowest temperature during pressing with the largest nozzle size in a screw press. On the contrary, phenolic compounds naturally present in BCS were less soluble in the oil phase and remained in BCC, causing a 1.5–2.5-fold increase in the TPC of BCC samples compared to oils (Table 2). Importantly, the most heated BCC obtained after pressing with the smallest nozzle size (5 mm) had the highest TPC (341 mg GAE/100 g), while the lowest TPC (284 mg GAE/100 g) was determined in the BCC produced using the largest nozzle size (10 mm) (Table 2 and Figure 3b). This can be explained by the fact that high temperatures of BCC (about 60 °C) can cause a cascade of transformations of phenolic compounds, such as their releasing from bound structures, chemical alteration, and changes in the enzymatic activity connected to cellular swelling and rupture, contributing to increasing amounts of more hydrophilic phenolic compounds, which did not transfer into the oil and remained in the by-product.

With reference to results listed in Table 2, the TPC in the BCC samples obtained after screw pressing with different nozzle sizes gave no statistically significant negative correlation (r = −0.9419, *p* = 0.2180) with their DPPH values. This indicates that a higher temperature of BCC increased the release of phenolic compounds, which were not necessarily effective in the DPPH radical inhibition.

Our results confirm that a change in nozzle diameter in the screw press generally affects the quality and antioxidant properties of both the final product (BCCPO) and by-product (BCC), which are free of chemicals and rich in bioactive compounds.

For comparison, Kiss et al. [19] reported an insignificant effect of nozzle diameters and rotational speed on the DPPH values (46.91–49.51 mg TEA/g, 44.78–50.59 mg TEA/g, and 46.51–49.68 mg TEA/g for 6, 8, and 10 mm of nozzle sizes, respectively) of oils pressed from walnut kernels, while TPC results (40.82–116.72 mg GAE/g) were higher in oils produced using a 6 mm nozzle size than those pressed with a nozzle size of 10 mm (TPC = 25.95–82.59 mg GAE/g). However, significantly higher values of DPPH (239.54–241.16 mg TEA/g, 207.46–211.81 mg TEA/g, and 205.58–227.72 mg TEA/g for 6, 8, and 10 mm of nozzle sizes, respectively) and TPC (157.57–221.58 mg GAE/g, 155.63–186.12 mg GAE/g, and 138.65–175.28 mg GAE/g for 6, 8, and 10 mm of nozzle sizes, respectively) were measured in walnut bagasse pellets than in pressed oils. Similar to our results, these authors found that a smaller nozzle diameter generated higher contents of phenolic compounds in bagasse pellets. In addition, a significant increase in antioxidant activity analyzed by the DPPH method (by 36%) and TPC (from 16.7 to 35.4 mg/kg) in virgin pistachio oils was observed when a wider nozzle diameter (6 mm) in a screw press was changed to a smaller nozzle diameter (3 mm) [14]. However, the higher phenolic content (TPC = 8600–14,864 mg/kg) and the corresponding antioxidant activity (DPPH = 18.94–46.12 mmol/kg) of pistachio residual cakes obtained using a 6 mm nozzle size, compared to the TPC (8037–10,764 mg/kg) and DPPH (15.45–33.24 mmol/kg) of residues after pressing with a 3 mm nozzle size, make these by-products, regardless of their processing conditions, a rich source of functional ingredients.

### 3.3. Effect of Acid and Alkaline Hydrolysis on Antioxidant Properties of Black Cumin Meal

**Figure 4:**
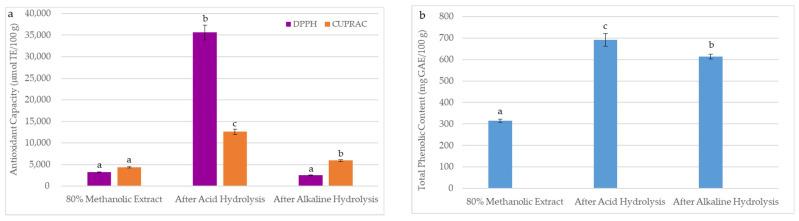
Effects of extraction methods (conventional mechanical extraction, acid hydrolysis, and alkaline hydrolysis) on (**a**) antioxidant capacity and (**b**) total phenolic content in black cumin meal. The DPPH, CUPRAC, and TPC were presented as mean (*n* = 5) with standard deviation (SD). Bars with different letters (a–c) represent significant differences between DPPH, CUPRAC, and TPC in black cumin meal samples (one-way ANOVA and Duncan test, *p* < 0.05).

The technological processes of oilseeds used to obtain oils generate large amounts of final residues after solvent extraction, called a meal. In recent years, the sustainability and valorization of by-products by extracting residual bioactive compounds have become crucial for the food industry. Due to the by-product quality reflecting the main product’s characteristics, the antioxidant properties of BCM after hydrolysis were studied. The effect of acid hydrolysis and alkaline hydrolysis on the DPPH, CUPRAC, and TPC in the prepared BCM hydrolysates is shown in Figure 4. As can be seen, DPPH, CUPRAC, and TPC in the investigated BCM samples were significantly higher after acid hydrolysis (DPPH = 35,629 µmol TE/100 g, CUPRAC = 12,601 µmol TE/100 g, TPC = 691 mg GAE/100 g) than after alkaline hydrolysis (DPPH = 2539 µmol TE/100 g, CUPRAC = 5959 µmol TE/100 g, TPC = 613 mg GAE/100 g). A higher release of antioxidants after acid hydrolysis is probably due to the presence of more phenolic compounds in the BCM forming glycosidic bonds, which break during acid treatment.

As depicted in Figure 4a, an insignificant lower DPPH value (2539 µmol TE/100 g) was observed for alkaline hydrolysate than the DPPH (3222 µmol TE/100 g) of the BCM extract prepared by the conventional extraction with 80% methanol. This suggests that a sodium hydroxide solution used for alkaline hydrolysis caused the degradation of some antioxidants capable of scavenging DPPH radicals. However, this reaction hydrolyzed the ester bonds and ether bonds between the phenolic compounds and the BCM cell wall, causing their escape and forming the orange Cu(I)-Nc complex (Figure 4a).

Furthermore, Figure 4b illustrates that alkaline hydrolysis was only 11% less effective than acid hydrolysis in releasing and extracting bound phenolics from BCM. However, total amounts of phenolics in acid (TPC = 691 mg GAE/100 g) and alkaline (TPC = 613 mg GAE/100 g) hydrolysates were approximately two and seven times higher than TPC in 80% and 100% of methanolic BCM extracts (315 and 92 mg GAE/100 g, respectively) (Table 1 and Figure 4).

For comparison, nonhydrolyzed methanolic extracts of *Nigella sativa* L. seeds had significantly lower concentration of total phenolics (TPC = 67 mg GAE/100 g) and antioxidant potential determined by the FRAP (24 mg GAE/100 g), DPPH (139 mg TE/100 g), and ABTS (103 mg TE/100 g) methods than antioxidant properties of alkaline and acid hydrolyzed extracts (TPC = 73 and 71 mg GAE/100 g, FRAP = 28 and 26 mg GAE/100 g, DPPH = 167 and 154 mg TE/100 g, ABTS = 112 and 109 mg TE/100 g, respectively) [36]. Contrary to our results, the acid-hydrolyzed extract had somewhat lower antioxidant properties in comparison with the alkaline-hydrolyzed extract. Moreover, Prakash et al. [31] reported significantly higher levels of phenolic compounds under alkaline hydrolysis (TPC = 1820.53–2015.28, 961.25–1251.35, and 251.50–396.69 mg GAE/100 g) than acid hydrolysis (TPC = 1155.19–1212.58, 731.25–857.21, and 117.50–178.45 mg GAE/100 g) in three nut by-products, cashew nut shell cake, coconut shell, and groundnut hull, respectively. Similarly, alkaline hydrolyzed extracts of olive pomace from various cultivars produced the highest phenolic content (TPC = 0.68–5.03 mg GAE/g) and antioxidant capacity (IC_50_ = 0.16–5.00 and 0.05–247.11 mg/mL for DPPH and ABTS assays, respectively, CUPRAC = 5.00–25.22 mg GAE/g, FRAP = 4.60–30.60 mg ascorbic acid (AA)/g), followed by extracts after acid hydrolysis (TPC = 1.00–4.00 mg GAE/g, IC_50_ = 0.31–7.79 and 0.03–574.30 mg/mL for DPPH and ABTS assays, respectively, CUPRAC = 6.33–15.47 mg GAE/g, FRAP = 5.91–20.93 mg AA/g) and the lowest TPC (0.02–2.27 mg GAE/g), DPPH (IC_50_ = 1.11–8.76 mg/mL), ABTS (IC_50_ = 0.27–1837.88 mg/mL), CUPRAC (5.00–15.00 mg GAE/g), and FRAP (0.57–10.74 mg AA/g) were found for extracts after maceration with methanol [28]. However, in agreement with our results, total amounts of phenolics and antioxidants with FRAP capacity, released during acid hydrolysis from brewers’ spent grains (TPC = 10.59 mg GAE/g, FRAP = 11.87 mmol Fe(II)/g), hazelnut shells (TPC = 7.92 mg GAE/g, FRAP = 16.71 mmol Fe(II)/g), and orange peels (TPC = 19.70 mg GAE/g, FRAP = 15.54 mmol Fe(II)/g), were higher than those after the alkaline hydrolysis (TPC = 6.53, 2.25, and 7.83 mg GAE/g and FRAP = 8.25, 15.01, and 7.91 mmol Fe(II)/g for brewers’ spent grains, hazelnut shells, and orange peel liquors, respectively) [34].

The obtained TPC and CUPRAC results confirm the discrepancies in the effectiveness of antioxidant extraction from BCM using solvents with different polarities. A higher polar 80% methanol was more efficient in the recovery of phenolic compounds and hydrophilic antioxidants with the ability to reduce the Cu(II)-Nc complex compared to 100% methanol (Figure 4 and Table 1). On the contrary, the 100% methanolic extract of BCM revealed a somewhat higher DPPH (3426 µmol TE/100 g) when compared to the DPPH value (3222 µmol TE/100 g) for an 80% methanolic extract. This can be explained by the fact that the FC assay detects hydrophilic phenolic compounds, the CUPRAC assay evaluates antioxidant mechanisms in both lipophilic and hydrophilic compounds, and the DPPH assay can only be applied to hydrophobic systems.

### 3.4. Greenness Evaluation of Oil Extraction Procedures

**Figure 5:**
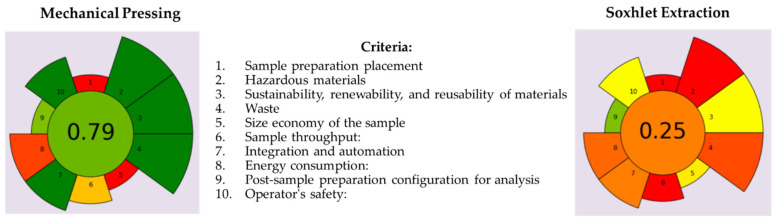
Comparison of greenness factors obtained for cold pressing and *n*-hexane extraction of black cumin oils from black cumin seeds. The total score ranked from 0 to 1 (the central, circular field corresponds to the final assessment score), and the 10 criteria were studied with a scaling of the criterion’s weight (1 to 5) against the total value in the form of segment size and value obtained on a scale from red to green.

Two conventional oil extraction methods from *Nigella sativa* L. seeds, such as cold pressing and solvent extraction, were evaluated in terms of their greenness factor using the AGREEprep metric as an emerging innovative tool. In this study, the AGREEprep calculator metric tool was focused on seed preparation for oil recovery and depended on 10 environmental impact criteria, depicted in Figure 5.

As can be seen, the final greenness scores of 0.79 and 0.25 calculated for the oil extraction by cold pressing and *n*-hexane extraction, respectively, differed significantly. Due to the fact that an overall score greater than 0.6 is considered indicative of a green method, the screw pressing production of BCCPO is the greenest methodology. The main drawback of both applied procedures was criterion 1 scored with 0 because the techniques required were carried out in a laboratory with adequate equipment (screw press and Soxhlet apparatus) after the transport of seed samples (ex situ). On the contrary, the solvent extraction of oil from BCS was based on the use of an organic, hazardous solvent (*n*-hexane), causing a significant decrease in score rating (0.00, 0.50, and 0.15) for criteria 2, 3, and 4. However, both oil extraction processes revealed a similar energy consumption on a laboratory scale, and the qualities of the obtained oils were characterized using simple spectrophotometric techniques, while the selection of cold pressing reduced process time. The application of screw pressing as a highly controlled technique for natural matrices without any toxic solvents favors the health of the operators, obtaining a 1.00 score for criterion 10. For comparison, this criterion was scored with 0.50 in the case of the Soxhlet method used to recover black cumin oil from *Nigella sativa* L. seeds because this technique required the use of *n*-hexane and solvent evaporation. Importantly, oil extraction methods were assessed for their greenness based on a laboratory-level study, but these processes need to be adjusted to an industrial scale.

## 4. Conclusions

The changes in antioxidant properties throughout the black cumin chain, from seeds to oils and by-products on each technological step, were analyzed in accordance with the consumers’ need for healthy vegetable oils with high natural antioxidants, that lower the risks of chronic diseases and increase life expectancy, while considering sustainability and the valorization of by-products rich in bioactive compounds.

Both conventional oil extraction methods, such as cold pressing and solvent extraction, widely used in the oil industry, significantly affected the antioxidant properties of cold-pressed black cumin oil and solvent-extracted black cumin oil, as well as two by-products, black cumin cake and black cumin meal, respectively. The solvent extraction method allowed the production of crude black cumin oil with the highest content of phenolic compounds and antioxidant potential determined by two analytical methods (DPPH and CUPRAC) based on different mechanisms. However, the antioxidant properties of cold-pressed black cumin oil were similar to those of black cumin cake, which is a by-product of cold pressing. The application of cold pressing as a greener oil extrusion method compared to the *n*-hexane extraction method confirmed that oil efficiency and the antioxidant properties of cold-pressed black cumin oils and residual cakes depended on using different nozzle diameters in a screw press. The use of a smaller nozzle diameter (5 mm) increased oil yield and temperature while reducing the number of antioxidants and hydroperoxide in cold-pressed black cumin oil (the lowest DPPH, CUPRAC, TPC, and PV results were found). It is likely that the high PV results for oils screw-pressed with different nozzle diameters can be related to the high amount of thymoquinone, which acts as an interfering substance during PV determination using the official iodometric method, leading to an overestimation of the PV results. Therefore, future studies are necessary to evaluate the effect of screw pressing conditions on thymoquinone concentration in the obtained black cumin oils and its consideration in the modified procedure of PV determination. Moreover, the antioxidant capacity of cold-pressed black cumin oils and residual cakes increased with increasing nozzle sizes. However, black cumin cake containing the highest number of total polyphenols, obtained after screw pressing with a nozzle size of 5 mm, had the lowest weight and oil content. In addition, acid hydrolysis breaking glycosidic bonds more effectively enhanced the number of phenolic compounds and the antioxidant capacity of black cumin meal extract compared to alkaline hydrolysis.

Despite some variabilities in studies focusing on oil extraction methods, the valorization of by-products such as black cumin cake and black cumin meal rich in antioxidants due to high DPPH, CUPRAC, and TPC values can be paramount for making a profit and offsetting the costs of advanced oil extraction technologies.

## Figures and Tables

**Table 1:** Antioxidant capacity and total phenolic content in black cumin seeds, oils, and by-products.

Samples	Antioxidant Capacity		TPC * ± SD (mg GAE/100 g)
	DPPH * ± SD	CUPRAC * ± SD	
	(µmol TE/100 g)		
Raw material			
BCS	1219 ± 46 ^a^	871 ± 39 ^a^	182 ± 13 ^c^
Oils			
BCCPO	3451 ± 89 ^b^	3475 ± 172 ^d^	259 ± 6 ^d^
BCEO-S	16,500 ± 249 ^d^	4827 ± 144 ^e^	292 ± 9 ^e^
BCEO-C	4041 ± 181 ^c^	1275 ± 44 ^b^	70 ± 1 ^a^
By-products			
BCC	3305 ± 127 ^b^	3397 ± 131 ^d^	426 ± 23 ^f^
BCM	3426 ± 63 ^b^	1514 ± 74 ^c^	92 ± 4 ^b^

* *n* = 5; SD—Standard Deviation; different letters (a–f) within the same column indicate significant differences between DPPH, CUPRAC, and TPC in the studied samples (one-way ANOVA and Duncan test, *p* < 0.05).

**Table 2:** Effect of nozzle diameters on quality and antioxidant capacity of cold-pressed black cumin oil and black cumin cake.

Parameter	Nozzle Diameters (mm)		
	5	8	10
Cold-Pressed Black Cumin Oil			
Oil yield * ± SD (%)	48.86 ± 0.40 ^c^	23.79 ± 0.29 ^b^	20.92 ± 0.23 ^a^
PV * ± SD (meq O_2_/kg)	74.92 ± 1.93 ^a^	79.77 ± 1.42 ^b^	84.38 ± 3.79 ^c^
DPPH * ± SD (µmol TE/100 g)	2933 ± 76 ^a^	4880 ± 902 ^b^	5894 ± 98 ^c^
TPC * ± SD (mg GAE/100 g)	135 ± 8 ^a^	145 ± 8 ^a^	199 ± 12 ^b^
Black Cumin Cake			
Weight of cake * ± SD (g/kg seeds)	511.40 ± 2.26 ^a^	762.10 ± 1.58 ^b^	790.76 ± 1.75 ^c^
Oil in cake * ± SD (%)	23.59 ± 0.46 ^a^	26.64 ± 0.44 ^b^	28.79 ± 0.08 ^c^
Water in cake * ± SD (%)	6.70 ± 0.03 ^a^	6.36 ± 0.24 ^a^	6.48 ± 0.17 ^a^
DPPH * ± SD (µmol TE/100 g)	1890 ± 151 ^a,b^	1958 ± 76 ^a,b^	2265 ± 381 ^b^
TPC * ± SD (mg GAE/100 g)	341 ± 12 ^c^	313 ± 14 ^b^	284 ± 17 ^a^

* *n* = 5; SD—Standard Deviation; different letters (a–c) within the same row indicate significant differences between determined parameters (one-way ANOVA and Duncan test, *p* < 0.05).

## Data Availability

The original contributions presented in the study are included in the article, further inquiries can be directed to the corresponding author.
